# Physiological and molecular implications of multiple abiotic stresses on yield and quality of rice

**DOI:** 10.3389/fpls.2022.996514

**Published:** 2023-01-11

**Authors:** Beena Radha, Nagenahalli Chandrappa Sunitha, Rameswar P. Sah, Md Azharudheen T. P., G. K. Krishna, Deepika Kumar Umesh, Sini Thomas, Chandrappa Anilkumar, Sameer Upadhyay, Awadhesh Kumar, Manikanta Ch L. N., Behera S., Bishnu Charan Marndi, Kadambot H. M. Siddique

**Affiliations:** ^1^ Department of Plant Physiology, Kerala Agricultural University-College of Agriculture, Vellayani, Thiruvananthapuram, Kerala, India; ^2^ Department of Genetics and Plant Breeding, University of Agricultural Sciences, Bangalore, Karnataka, India; ^3^ Division of Crop Production, Indian Council of Agricultural Research-National Rice Research Institute, Cuttack, Odisha, India; ^4^ Department of Plant Physiology, Kerala Agricultural University-College of Agriculture, Thrissur, Kerala, India; ^5^ Mulberry Breeding & Genetics Section, Central Sericultural Research and Training Institute-Berhampore, Central Silk Board, Murshidabad, West Bengal, India; ^6^ Department of Plant Physiology, Kerala Agricultural University-Regional Agricultural Research Station, Kumarakom, Kerala, India; ^7^ Department of Plant Physiology, Indira Gandhi Krishi Vishwavidyalaya, Raipur, India; ^8^ The University of Western Australia Institute of Agriculture, The University of Western Australia, Perth, WA, Australia

**Keywords:** multiple abiotic stresses, physiology, high temperature, salinity, drought, eCO2, sensitivity, tolerance

## Abstract

Abiotic stresses adversely affect rice yield and productivity, especially under the changing climatic scenario. Exposure to multiple abiotic stresses acting together aggravates these effects. The projected increase in global temperatures, rainfall variability, and salinity will increase the frequency and intensity of multiple abiotic stresses. These abiotic stresses affect paddy physiology and deteriorate grain quality, especially milling quality and cooking characteristics. Understanding the molecular and physiological mechanisms behind grain quality reduction under multiple abiotic stresses is needed to breed cultivars that can tolerate multiple abiotic stresses. This review summarizes the combined effect of various stresses on rice physiology, focusing on grain quality parameters and yield traits, and discusses strategies for improving grain quality parameters using high-throughput phenotyping with *omics* approaches.

## 1 Introduction

Global warming and accompanying climate variabilities adversely impact global agricultural output, dwindling the production of food grains such as rice (Ramegowda and Senthil-Kumar, 2015). Abiotic stresses such as heat or temperature stress, submergence, drought, or nutritional deficiency create suboptimal environments (Jeyasri et al., 2021) that impair germination, seedling establishment, vegetative growth, flower initiation, panicle growth, grain filling, and productivity (Banerjee and Roychoudhury, 2020; Beena et al., 2021a). In rice, these attributes severely compromise crop establishment, growth (Beena et al., 2021b; Anie et al., 2022; Stephen et al., 2022), grain quality, and productivity (Pravallika et al., 2020; Pathak et al., 2021). Some abiotic pressures in rice-growing environments spur the development and infection of biotic causal agents, aggravating the losses in productivity (Narsai and Whelan, 2013) and grain quality.

As the major staple food crop in the world, reductions in rice production due to climate change will have serious socioeconomic impacts. Many paddy growers experience frequent crop failure, resulting in unprecedented hardships such as starvation and financial pressure (Rejeth et al., 2020). Exposure to multiple abiotic stresses leads to physical and biochemical alterations in crop produce (Manikanta et al., 2020; Ali et al., 2022; Manikanta et al., 2022). Concurrent abiotic stresses damage rice crops more than individual stresses (Pandey et al., 2017), posing various physiological effects that trigger cross-talk reactions that affect rice phenology (Ramu et al., 2016; Ali et al., 2022). While not an abiotic stress component, elevated CO_2_ (eCO_2_) can alleviate or aggravate the stress effects.

Rice grain quality is measured primarily on the physical appearance of the grain, mineral content, proportion of amylose and amylopectin starch, aroma, and cooking quality (Chakraborti et al., 2021). Abiotic stresses during grain filling affect milling quality, grain chalkiness, starch composition, and cooking quality (Lanning and Siebenmorgen, 2013). According to ; Liu et al. (2021), high-temperature stress has the greatest impact on grain quality attributes, including reducing the sensory qualities of milled rice. Numerous studies have investigated the fundamentals of rice grain biochemistry, but few have examined how multiple abiotic stresses affect grain quality (Liu et al., 2013; Kadam et al., 2014).

Among abiotic stresses, high temperatures are particularly devastating, decreasing productivity and grain biochemical components. High temperatures decrease photosynthesis and photorespiration, decreasing total biomass production (Moore et al., 2021). High temperatures post-anthesis affect grain quality and appearance and decrease grain production (Dong et al., 2014). Similarly, high temperatures reduce pollen viability and increase spikelet sterility, decreasing grain production and quality (Rang et al., 2011). Extreme temperature stress at the maturity stage abates grain chalkiness, physical appearance, and biochemical properties such as amylose content and protein composition (Ahmed et al., 2015).

Excessive water stresses such as waterlogging and submergence adversely affect rice growth and grain yield. While some historical rice cultivars exhibit notable resilience to submergence, their total yield suffers (Singh et al., 2014). In contrast, modern rice cultivars are sensitive to flooding, often resulting in farmers losing their whole crop. Rice plants can perish soon after flooding due to high energy expenditure and protein hydrolysis during submergence. Flooding degrades the quality of endosperm reserves, adversely affecting the nutritional value and milling and cooking properties of rice grain (Zhou et al., 2020). Flooding at harvest-ready stages results in pre-harvest sprouting, compromising the marketable grain quality (Nonogaki et al., 2018) and reducing the grain’s eating and cooking quality (Zhou et al., 2020).

In recent decades, rice researchers have been working to improve crop yield and quality under stressful situations (Patra et al., 2020). Genomic techniques have been used to investigate how abiotic stresses affect grain development (Verma et al., 2021), with several genetic regulators of tolerance identified and successfully used to improve rice cultivars. For example, genetic loci controlling salinity stress have been discovered and pyramided to develop green super rice types (Pang et al., 2017). Using marker-assisted breeding, Kumar et al. (2018) combined quantitative trait loci (QTL) for submergence and drought tolerance to identify varieties with high yield potential, validating their performance by exposing them to various stresses. However, little information is available on combining stress tolerance and grain quality traits to fulfill food security (Ali et al., 2021).

Another major concern affecting plant growth is eCO_2_, with carbon dioxide levels expected to reach 685 ppm by 2050, raising the global mean temperature by 3–6°C relative to the pre-industrial era (Kilkis and Caglar, 2022). At the global level, crop models suggest that eCO_2_ levels could increase precipitation, but large spatial and temporal variabilities exist at the regional scale. Rainfall occurrence and intensity can be unpredictable, creating patches of drought and waterlogging (Walter, 2018). Various experiments have indicated that optimum levels of eCO_2_ can mitigate the effects of drought stress.

Candidate gene markers can be used to identify genes or QTL for grain production (Azharudheen et al., 2022). Anabolic gene expression requires favorable environmental conditions. Increased temperature impairs starch production, slowing sugar and starch metabolism and thus reducing grain filling and the number of filled grains per panicle (Fahad et al., 2019); a similar response occurs under salt stress (Hussain et al., 2017). Furthermore, significant QTL identified for drought tolerance are crucial for normal reproduction in paddy under drought (Catolos et al., 2017; Feng et al., 2018). The effects of combined mild salinity stress (75 mM NaCl) and moderately high temperatures (30/26°C day/night) were not additive when compared to the individual stresses. The combined stress had longer seedling roots and higher relative water content and Chl b than the salinity treatment alone. He et al. (2018) reported that ABA treatment mimicked protein perturbations in rice subjected to combined salinity stress and desiccation. In another study, Wytynck et al., 2021 reported similar ultrastructural changes in young leaf cells of rice seedlings subjected to salinity or high temperature stress, including the enhanced formation of rough endoplasmic reticulum assembly, reduced cristae formation in mitochondria, and disorganized cell wall fibrils.

QTL conferring tolerance to drought (*qDTY1.1, qDTY2.1*), salinity (Saltol), and submergence (Sub1) were introgressed by marker-assisted breeding, resulting in a climate-ready rice genotype, Improved White Ponni, a classic example of how information from multiple studies can assist in pyramiding traits for crop improvement (Muthu et al., 2020). The basal methylation patterns in the genomes of Pokkali (salinity tolerant), Nagina 22 (drought tolerant), and IR64 (susceptible) revealed that various stress-associated transcription factors (TFs) and signaling intermediates hypermethylated and thus downregulated to impart stress tolerance relative to IR64 (Garg et al., 2015). In addition, submergence-tolerant rice (FR13A) could withstand the compromise in photosynthetic traits despite lacking innate salinity tolerance (Sarkar et al., 2016). Several combined salinity and submergence stress experiments have revealed various physiological responses in rice. In one study, one week of this combined stress had little impact, while two weeks had detrimental effects on paddy rice, decreasing the relative growth rate, increasing the time to flowering, and decreasing yield (Kurniasih et al., 2021).

This review investigates the individual and interactive effects of various abiotic stresses (e.g., drought, salinity, high temperature, eCO_2_, submergence, nutrient deficiency) on rice growth, agronomy, and physiological traits, including grain quality and production, and the benefits of genomics for improving rice productivity and grain quality.

## 2 Physiological and molecular implications of individual stresses in rice yield and quality

### 2.1 Impact of drought stress on paddy

Climate change disrupts the regularity and magnitude of hydrological events, threatening crop production and affecting food security. The major regions affected include South and Southeast Asia, Sub-Saharan Africa, and Latin America, with unbunded and bunded uplands and shallow rainfed lowlands. Globally, drought stress events account for up to 40% of overall crop and livestock output losses, totaling nearly USD 28 billion (FAO, 2017). In Asia, frequent drought stress affects about 34 million ha of rainfed lowland rice and 8 million ha of upland rice (Barik et al., 2019). Drought stress frequently affects an area of 27 million ha of rainfed rice area (Shamsudin et al., 2016). In 2002, severe drought and depleted soil moisture affected over 65% of South Asia, resulting in considerable rice yield losses (~400 kg ha^–1^).

Water deficit causes numerous unfavorable changes in rice (Nithya et al., 2020). For example, 15 days of drought stress during reproductive stage reduced rice yields by up to 70%, increasing to up to 88% during flowering and 52% during grain filling. Drought stress at the flowering stage resulted in incomplete panicle exertion, 30% spikelet sterility, and a 20–46% reduction in seed set in a set of rice cultivars (Bahuguna et al., 2018). Drought stress during grain filling stage increases the proportion of chalky grains (Yang et al., 2018). The imposition of drought stress at the onset of anthesis for 30 days reduced the grain yield and harvest index of 25 rice genotypes, with reduced pollen fertility and test weight of grains for most genotypes, compared to irrigated conditions (Ahmad et al., 2022). While leaf rolling is considered a defense mechanism against drought stress, its promptness correlated with anatomical traits rather than water deficit (Nithya et al., 2021. While more leaf rolling occurred in genotypes such as Dangar, water deficit did not affect transpiration (Cal et al., 2019). Drought stress also affects the root system, with the ill-effects on root architecture and yield genotype-dependent (Prince et al., 2015; Beena et al., 2017; Beena et al., 2018c).

Plants have developed numerous adaptive responses to drought stress that aid their survival, including deeper roots, reduced water loss from shoots due to thick cuticle deposition, reduced leaf area, and osmotic adjustment, primarily by maintaining a high internal water status (Beena et al., 2018b; Manikanta et al., 2020; Rejeth et al., 2020). Beena et al. (2012) reported that root architecture, water uptake, and osmotic adjustment are important traits for drought tolerance screening. Physiological and biochemical changes in rice under drought are given in **Supplementary Table 1**. In rice, QTL mapping has revealed regions responsible for physiological traits, yield, and yield components. **Table 1** lists QTL/genes introgressed into rice for drought stress tolerance.

**Table 1:** Major QTLs reported for physio-morphological traits under various abiotic stress conditions in rice.

Trait	QTLs/Genes	Chromosome		Flanking markers	References
High yield under drought deployed for introgression using MAS in rice					
High yield under drought condition	*qDTY1.1*	1		RM431–RM104	Ghimire et al. (2012)
		1		RM104–RM12091	
		1		RM11943–RM12091	Vikram et al. (2011)
		1		RM486–RM472	Venuprasad et al. (2012)
		1		RM472	Muthu et al. (2020)
	*qDTY1.3*	1		RM488–RM315	Sandhu et al. (2014)
	*qDTY1.2*	1		RM259–RM315	
	*qDTY2.1*	2		RM2634	Muthu et al. (2020)
	*qDTY2.2*	2		RM236–RM279	Swamy BP. et al. (2013)
		2		RM211–RM263	Sandhu et al. (2014)
		2		RM211–233A	Palanog et al. (2014)
	*qDTY2.3*	2		RM263–RM573	Sandhu et al. (2014)
		2		RM573–RM250	Palanog et al. (2014)
		3		RM168–RM468	Dixit et al. (2014)
	*qDTY3.2*	3		RM569–RM517	Yadaw et al. (2013)
		3		RM60–RM22	Vikram et al. (2011)
	*qDTY4.1*	4		RM551–RM16368	Swamy BP. et al. (2013)
	*qDTY6.1*	6		RM589–RM204	Venuprasad et al. (2012)
		6		RM589-RM204	
		6		RM586-RM217	Dixit et al. (2014)
	*qDTY6.2*	6		RM121-RM541	Dixit et al. (2014)
	*qDTY9.1*.	9		RM105-RN434	Swamy BP. et al. (2013)
	*qDTY10.1*	10		RM216–RM304	Vikram et al. (2011)
	*qDTY10.2*	10		RM269–G2155	Swamy BP. et al. (2013)
	*qDTY12.1*	12		RM28166–RM28199	Mishra et al. (2013)
Submergence					
High survival	qSUB1.1	1		id1000556-id1003559	Gonzaga et al. (2016)
High survival	qSUB4.1	4		id4010621-id4012434	Gonzaga et al. (2016)
High survival	qSUB8.1	8		id08005815-id8007472	Gonzaga et al. (2016)
High survival	qSUB10.1	10		id10005538-RM25835	Gonzaga et al. (2016)
Anaerobic germination	qAG-5	5		RM405–RM249	Jiang et al. (2006)
Anaerobic germination	qAG-7-2	7		RM21868-RM172, seq- rs3583	Angaji et al. (2010); Zhang et al. (2017)
Anaerobic germination	qAG-7-1, AG2	7		RM3583–RM21427	Septiningsih et al. (2013)
Anaerobic germination	qAG-9-2, AG1	9		RM3769-RM105, seq- rs4216	Angaji et al. (2010); Zhang et al. (2017)
Anaerobic germination	qAG-11	11		RM21–RM22, seq-rs5125	Angaji et al. (2010); Zhang et al. (2017)
Anaerobic germination	qAG-1-2	1		RM11125-RM104; id29187939id32847451	Angaji et al. (2010); Hsu and Tung (2015)
Anaerobic germination		3		RM7097-RM520	Angaji et al. (2010)
Anaerobic germination	qAG-9-1	9		RM8303-RM5526	Angaji et al. (2010)
High survival	*qSUB8.1*	8		8,608,433–8,686,009	Gonzaga et al. (2017)
High survival	*qSUB2.1*	2		2,430,179–2,470,790	Gonzaga et al. (2017)
Salinity					
Na^+^ absorption/Na^+^ uptake	*qSNK1*	1		RM1287-RM10825	Thomson et al., 2010
	*qSNK2*	2		2422788 – 2437583*	Gimhani et al., 2016
	*qSNK4.1*	4		4355198 – 4384860*
	*qNaK3.1*	3		RM282-RM156	Puram et al., 2018
	snkr1.1	1		RM1287-AP3206d	de Ocampo et al., 2022
	*qNaK-R1.1*	1		RM472-RM14	Rahman et al., 2019
	*qNaK-R3.3*	3		RM5626- R3M53
	*qNaK-R5.4*	5		RM163-RM19199
Relative shoot potassium conc. compared to control	*qSRI-K9.1*	9		RM296-RM105	Puram et al., 2018
	*qSRI-NaK9.1*	9		RM296-RM105
Na^+^/K^+^ ratio in root	*qNa/KR-9*	9		HvSSR09-11-HvSSR09-39	Pundir et al., 2021
	*qRNK1*	1		RM1287-RM10825	Thomson et al., 2010
Na^+^/K^+^ ratio in leaf	*qNa/KL-1.3*	1		HvSSR01-56HvSSR01-70	Pundir et al., 2021
Na^+^/K^+^ ratio in leaf at reproductive stage	*qNa+/K+LR-3.1*	3		RM563-RM186	
Root Na^+^/K^+^ ratio	*qRNK1*	1		RM1287-RM10825	Thomson et al., 2010
	*qSNC1*	1		RM1287-RM10793	Thomson et al., 2010
	*qSNC-12*	12		RM1285-RM423	Zheng et al., 2015
	*qSNC3*	3		3528886– id3017899*	Gimhani et al., 2016
	*qSNC10*	10		9898598 – id10000153*
	*qNa3.3*	3		RM5626-R3M53	Rahman et al., 2019
Na^+^ in leaves at vegetative stage	*qNa+LV-8.2*	8		RM3395-RM281	
Na^+^ in leaves at reproductive stage	*qNa+LR-8.1*	8		RM3395-RM281
Na^+^ conc.in leaf	*qNaL-1.2*	1		HvSSR01-56HvSSR01-70	Pundir et al., 2021
Shoot K^+^ Conc	*Trait based QTL*	12		G24-R1684	Lang et al., 2001
	*Trait based QTL*	1		G24-R1684	Koyama et al., 2001
	*qSKC1*	1		RM8094-RM10825	Thomson et al., 2010
	*qSKC-2*	2		RM1285-RM423	Zheng et al., 2015
	*qSKC10*	10		13069784 – 9922981*	Gimhani et al., 2016
	*qK-6*	6		RM3827-RM340	Sabouri et al., 2009
	*qK3.2*	3		RM5626-R3M53	Rahman et al., 2019
	*qK12.3*	12		RM27615-RM27877
	*qK3.1*	3		RM282-RM156	Puram et al., 2018
Root Na^+^ content	*qRNC-9*	9		RM201-RM215	Zheng et al., 2015
	*qNaR-9*	9		HvSSR09-11-HvSSR09-39	Pundir et al., 2021
	rnc3.1	3		SO3072-SO3099	de Ocampo et al., 2022
Root K^+^ Conc	*qRKC-4*	4		C891-C513	Lin et al., 2004
	*qRKC1*	1		RM1287-RM11330	Thomson et al., 2010
	*qRKC6*	6		RM19840-RM20350
	*qKR-1*	1		HvSSR01-11-HvSSR01-34	Pundir et al., 2021
	*qKR-12*	12		HvSSR12-11-HvSSR12-28
	*qKR-7.1*	7		HvSSR07-25-HvSSR07-37
	*rkc3.1*	3		SO3072-SO3099	de Ocampo et al., 2022
	*qSGEM-7*	7		CDO59-RG477	
Seedling dry matter	*qSDM-5*	5		RZ70-RZ225
	*qSDM-6*	6		CDO544-Amy2A
	*qSDM-10*	10		RZ625-RZ500
Seedling root length	qSRTL-6	6		RG162-RG653
Seedling height	*qSH1.2*	1		RM5389-RM5759	Wang et al., 2012
	*qSH1.3*	1		RM3482-RM3362
	*Trait based QTL*	7		C1057-R565
	*qSL1.3*	1		id1023892–id1017885*	Rahman et al., 2017
	*qSL5.3*	5		RM163-RM19199
	*qSHL4.2*	4		RM3866-RM3288	Puram et al., 2018
	*qSHL-5*	5		RM13-RM164	Ghomi et al., 2013
Shoot Fresh weight	*qFWsht1.2*	1		id1023892 –id1017885*	Rahman et al., 2017
	*qFWsht6.1*	6		id6016941–id6001397*
	*qSFW-5b*	5		RM459-RM3800	Ghomi et al., 2013
	*qDSW6.1*	6		RM6818-RM6811	Wang et al., 2012
	*qDSW6.2*	6		RM340-RM3509
	*qDWsht5.1*	5		id5007714–id5014589*
	*qDWT8.1*	8		RM44-RM515	Puram et al., 2018
	*qSDW-2*	2		RM279-RM5911	Ghomi et al., 2013
Root fresh weight	*qRFW-4b*	4		E36-M59-5E37-M60-3	Ghomi et al., 2013
	*rdw1.2*	1		RM11570-S01132A	de Ocampo et al., 2022
	*qRL-9*	9		RM219-RM7038	Zheng et al., 2015
	*rl2.1*	2		RM13332-RM5404	de Ocampo et al., 2022
Plant height	*qPH2*	2		RM13197-RM6318	Thomson et al., 2010
	*qSTR-3a*	3		RM1022-RM6283	
Visual tolerance score	*qSES-2*	2		RM1285-RM423	Zheng et al., 2015
Standard Evaluation	*qSES1.1*	1		ud1000711– Id1004348*	Rahman et al., 2017
	*qSES1.3*	1		id1024972– id1023892*	Gimhani et al., 2016
Overall Phenotypic performance	*qSES3.1*	3		RM5626- R3M53	Rahman et al., 2017
	*qSES5.2*	5		RM163-RM19199	Rahman et al., 2019
Survival %	*qSur1.1*	1		RM472-RM14	Puram et al., 2018
	*qSTR-3a*	3		RM1022-RM6283	
Salt survival index	*qSSI4.2*	4		454365 – 24572241*	Zheng et al., 2015 * SNPs were used
	*qSSI10*	10		9898598 – id10000153*
Panicle length	*qPL-2*	2		HvSSR02-66-HvSSR02-68	Rahman et al., 2019
Biomass	*qBM-8*	8		HvSSR08-11-HvSSR08-15
	*qBM-5a*	5		E36-M59-10-RM440	Ghomi et al., 2013
**High temperature**					
1. Spikelet fertility 2. Daily flowering time 3. Spikelet fertility and pollen shedding	*qSF^ht^2*, *qSF^ht^4.2* *qDFT3, qDFT8, qDFT10.1*, *qDFT11* *qPSL^ht^1*, *qPSL^ht^4.1*, *qPSL^ht^5*, *qPSL^ht^7*, *qPSL^ht^10.2*	2.4 3,8,10, 11 1,4,5,7,10		RM1234–RM3850, RM3916–RM2431 RM3766–RM3513 RM5891–RM4997 RM6737–RM6673 RM1355–RM2191 RM1196–RM6581 RM7585–Bb38P21 RM1248–RM4915 RM6394–RM1364 RM7492–RM1859	Zhao et al., 2016
Flowering time HT QTL	*qHTT8*	8		*LOC_Os08g07010* *LOC_Os08g07440*	Chen et al., 2021
1. Vegetative stage root length QTL 2. Vegetative stage root length QTL	*rlc1.1* *rlc1.2* *rlc4.1* *rlc4.2* *rlc4.3* *rlc7.1* *slc6.1* *slc6.2*	1,2,.3 1,2		S1_10221082 S1_30191377 S4_100099 S4_1911293 S4_13167045 S7_24934857 S6_9368784 S6_32050861	Kilasi et al., 2018
1. Filled grain number per panicle 2. Grain yield 3. HT Score				RM468 - RM7076 RM241 - RM26212 RM16686 - RM564 RM241 - RM26212 RM26212 - RM127 RM3586 - RM160	Buu et al., 2014
1. Spikelet sterility % 2. Yield per plant	*qSTIPSS9.1* *qSTIY5.1*	1,5			Shanmugavadivel et al., 2017
1. Spikelet fertility %	*qHTSF4.1*	4			Ye et al., 2015
1. Spikelet fertility %	*qHTSF1.2* *qHTSF2.1* *qHTSF3.1*	2,1,3			Ye et al., 2015
1. Spikelet fertility %	*qHTSF6.1* *qHTSF11.2*	6,11			Ye et al., 2015

* represents Single NucleotidePolymorphisms (SNPs).

### 2.2 Impacts of submergence on paddy

Rice is adapted to stagnant conditions because its well-developed aerenchyma promotes oxygen transport through roots. However, submergence caused by recurrent flooding can adversely affect plant growth and productivity. In lowland and deep-water rice areas, flooding occurs on more than 16 million ha, with annual economic losses estimated to exceed $600 USD million (www.knowledgebank.irri.org). In addition, unpredictable flash floods can occur at any stage of paddy development.

Submergence reduces the quality and quantity of rice, especially when it occurs during the reproductive and maturity stages. Submergence significantly delays flowering and maturity, reducing grain yield, shoot biomass, harvest index, and yield components (Marndi et al., 2022). Reductions in grain filling, grain number per panicle, and grain weight are primarily responsible for decreased grain production due to submergence (Kato et al., 2014). Submergence during the vegetative stage affects critical grain quality parameters, with a higher proportion of hull, brown rice, and bran in rough rice compared to non-stressed counterparts, as well as chalky grains, breakage during hulling, and reduced proportion of amylose, but increased in crude protein content. Starch accumulation negatively correlated with ADP-glucose pyrophosphorylase activity in submerged rice. ADP-glucose pyrophosphorylase (AGPase) catalyzes the first committed reaction in the pathway of starch synthesis. ADP-glucose pyrophosphorylase is activated by posttranslational redox-modification in response to light and to sugars in leaves of wheat and other plant species (Ferrero et al., 2020).

Yield losses due to submergence are attributable to a smaller sink size/capacity and reduced carbohydrate metabolism and thus reduced partitioning into grain. Djali et al. (2012) reported that submerged rice had higher protein, moisture, and amylase contents than the control plants but lower yield, hardness, stickiness, and brightness. Physiological and biochemical changes in rice under submergence/flash flooding is given in **Supplementary Table 1**. Further, increased starch and non-structural carbohydrate accumulation positively correlated with survival percentage under submerged conditions (Panda and Sarkar, 2014). **Table 1** lists QTL/genes identified in rice for submergence tolerance.

### 2.3 Impact of salt stress on paddy

Rice is sensitive to soil salinity, which occurs in 25–30% of irrigated regions of rice, equating to more than 1 billion ha of saline or sodic land (Shahid et al., 2018). Rice is more resistant to salt during the germination and vegetative stages than the seedling and reproductive stages. High-yielding rice cultivars at salinity levels >3 dS m^–1^ suffered yield losses of ~12%, which increased to ~50% at 6 dS m^–1^ (Kumar and Sharma, 2020). Plants subjected to salt stress have delayed seed germination and seed set, sterile spikelets, and reduced leaf dry matter, leaf area, tiller number, grains per panicle, pollen viability (Reshma et al., 2021).

Salt-stressed rice plants suffer from a reduced water potential, poor nutrient uptake, and increased sodium (Na^+^) and chlorine (Cl^–^) uptake. Salinity stress also affects proline and anthocyanin contents, peroxidase activity, and Ca^2+^, Na^+^, K^+^, chlorophyll, and H_2_O_2_ concentrations (Negrão et al., 2017). Salt stress significantly reduced amylose concentration in a salt-tolerant rice genotype but not a semi-tolerant genotype, even at low EC (4 mS cm^–1^) and alkalinity (pH 9.5), while high EC (8 dS m^–1^) and alkalinity (pH 9.8) significantly reduced starch content in both genotypes, but not the susceptible genotype (Rao et al., 2013). Details of physiological and biochemical changes in rice under salinity is listed in **Supplementary Table 1**. In addition, salinity (EC 4 and 8 mS/cm) and high alkalinity (pH 9.8) affected gel consistency in the salt-susceptible genotype (Rao et al., 2013). **Table 1** lists QTL/genes identified in rice for salinity-related traits.

### 2.4 Impact of high temperature on paddy

Heat stress in rice is related to specific morphological, physiological, biochemical, and molecular changes. Morphological aspects include genotypes that shield the panicles with their foliage to maintain a lower spikelet temperature for increased spikelet fertility (Beena et al., 2018a). An early morning flowering habit also plays a vital role in plants avoiding high temperatures later in the day (Hirabayashi et al., 2015; Raghunath and Beena, 2021).

Physiological mechanisms that provide heat stress tolerance in rice include an increased membrane stability index, which reduces reactive oxygen species (ROS) damage to biological membranes (Kumar et al., 2016). Increased pollen viability ensures increased fertilization success, maintaining a higher photosynthetic rate to offset yield losses due to excess transpiration rate under heat stress (Sinha et al., 2022). An increased transpiration rate ensures transpirational cooling to prevent ROS increases (Xiong et al., 2014). Physiological adaptations play a critical role in protecting membrane integrity and the biological compounds required to maintain cellular homeostasis. Heat shock proteins (HSPs), which maintain the tertiary structure of proteins, are also critical players in cellular tolerance (Khan and Shahwar, 2020). In addition, enzymatic and non-enzymatic antioxidants such as superoxide dismutase (SOD), peroxidase (POD), glutathione peroxidase (GPX), catalase (CAT), ascorbic acid, phenolic compounds, and carotenoids are crucial for negating the toxic effects of ROS (Irato and Santovito, 2021). Physiological and biochemical changes in rice under high temperature stress is given in **Supplementary Table 1**.

Marker-assisted introgression of QTL controlling spikelet fertility (Vivitha et al., 2018) and early morning anthesis traits (Ishimaru et al., 2022) under high-temperature conditions have contributed greatly to crop improvement. **Table 1** lists QTL/genes identified for physiological and yield traits in rice under high-temperature stress.

### 2.5 Impact of elevated CO_2_ on paddy

CO_2_ levels have risen from 270 ppm during the pre-industrial era (1850s) to 400 ppm. At this rate, atmospheric CO_2_ (aCO_2_) will reach eCO_2_ levels by 2050, estimated at 550 ppm, affecting the morphology, physiology and biochemistry of rice (Abdelhakim et al., 2022). A meta-analysis involving 125 studies on the effect of eCO_2_ in rice showed that hybrid cultivars respond with higher biomass and yield over popular *indica* and japonica types, primarily due to increased panicle and spikelet numbers, followed by tiller number. eCO_2_ levels increase the accumulation of root biomass more than shoot biomass (Wang et al., 2018). A three-year experiment in a free-air CO_2_ enrichment (FACE) facility revealed a declining proportion of brown, milled, and head rice under eCO_2_ (200 ppm above ambient) relative to aCO_2_ (Gao et al., 2021). In addition, the eCO_2_ increased grain chalkiness, viscosity, and stickiness but, improving palatability; however, the eCO_2_ compromised the processing quality and nutritional attributes such as protein and mineral contents (Ca, Cu and S; except for K) (Gao et al., 2021). A comparative study at eCO_2_ (700 ppm) improved seedling emergence, C/N ratio, and biomass in two rice genotypes (IR20 and ADT46). Changes in physiological traits under elevated CO_2_ is given in **Supplementary Table 1**. When subjected to brown plant hopper infestation, the eCO_2_-grown plants had greater insect attack, but insect survival decreased by several days, relative to the control plants (SenthilNathan, 2021). Thus eCO_2_ poses several ecological effects on rice-based agri-ecosystem.

### 2.6 Impact of soil nutrient deficit on paddy

Since the green revolution, fertilizer application is essential due to the unintentional emergence of fertilizer-responsive, high-yielding semi-dwarf rice cultivars (Neeraja et al., 2021). Reported poor nutrient use efficiencies in rice, with 30−50% for nitrogen, 30% for phosphorous, and 26% for potassium. In addition to macronutrients, breeders are now paying close attention to micronutrient deficits (‘hidden hunger’) due to human health concerns. The most common micronutrient disorders are Fe insufficiency, Zn deficiency, and B toxicity for wetland rice and Fe and B deficiency and Mn toxicity for upland rice (Shrestha et al., 2020).

Rice is the primary source of nutrition for much of the world’s population. However, rice is deficient in essential fatty acids, vitamins, minerals, phytochemicals, and amino acids (Sultana et al., 2022). Zhou et al. (2018) reported positive effects of nitrogen on the milling and nutritional quality of rice. Increased nitrogen application increased protein content but decreased milling quality, appearance, amylose content, gel consistency, cooking/eating quality, and rice flour viscosity (Zhu et al., 2017). The nitrogen‐efficient line (*OsNRT2*.3b‐overexpressing (O8) and wild type (WT) were treated with different levels of nitrogen and carbon fertilizers under field conditions to study the effects of different fertilization treatments on rice quality. The results showed that the appearance, nutrition, and taste qualities of O8 were generally high compared with WT under various fertilization treatment conditions (Zhang et al., 2022).

Rice is particularly vulnerable to nutrient deficit stress at the seedling emergence, tillering, panicle initiation, booting, heading, and maturity stages (Shrestha et al., 2020). During the early and mid-phases of grain filling, K and Ca control root exudation, which affects grain quality characteristics such as the proportion of chalky kernels, chalkiness, and amylose content (Lijun et al., 2011). N fertilization can affect micronutrient concentrations.

## 3 Physiological and molecular implications of combined abiotic stresses on rice yield and quality

### 3.1 Effect of combined drought and temperature

**Figure 1:**
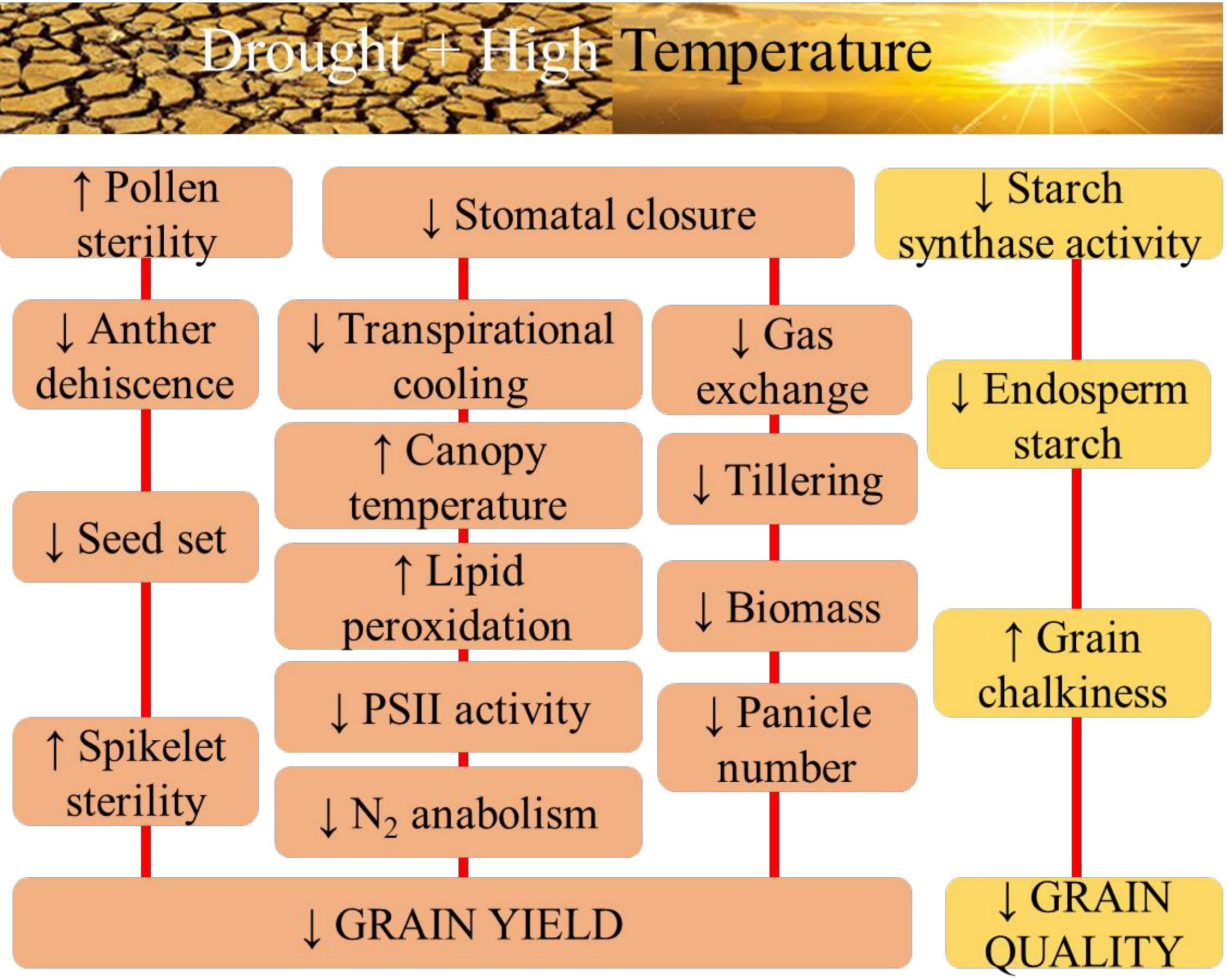
The physiological aspects of sensitivity to combined drought and high temperature stress in rice with respect to grain yield, and quality.

Drought and high-temperature stress often occur simultaneously in the field, drastically affecting plant growth, development, and yield by inducing physiological, biochemical, and molecular changes and responses that impact various cellular and whole plant functions (**Figure 1**). Combined effect of drought and high temperature is more severe than individual effects (Dreesen et al., 2012).

#### 3.1.1 Physiological and genetic components of sensitivity

Drought and heat stress combined affect rice crops at the cell, organ, plant, and canopy level, ultimately reducing growth and yield. The combined stress often has conflicting or antagonistic responses dissimilar to their individual effects. Vapor pressure deficit (VPD) naturally increases during heat waves and droughts, impacting rice physiology (Williams et al., 2014). During heat stress, plants open stomata to cool their leaves by transpiration but cannot open them when faced with combined heat and drought stress (Sinha et al., 2022). In perennial grasses, combined heat and drought stress reduces PSII function, weakens N anabolism, strengthens protein catabolism, and increases lipid peroxidation. Long-term combined heat and drought stress affects growth, leaf gas exchange, and water use efficiency (WUE) in rice, severely reducing total biomass relative to individual stresses (Perdomo et al., 2015; Perdomo et al., 2016).

Rice is more sensitive to drought, heat, and combined stress during the reproductive stage, specifically flowering, than the vegetative stage. Combined heat and drought stress at the seedling and tillering stages resulted in the absence of panicles for seven African rice cultivars (Mukamuhirwa et al., 2019). The number of germinated pollens on the stigma decreased when exposed to heat (81%), drought (59%) and concomitant stress (Rang et al., 2011). Combined heat and drought stress at flowering significantly affected peduncle length, anther dehiscence, pollen number, pollen germination, spikelet fertility, and thus yield in rice (Li et al., 2015; Rang et al., 2010). Heat and drought stress hinder the accumulation of various seed constituents in rice by inhibiting starch processes and protein synthesis. Grain quality is more susceptible to combined stress than yield. High temperature (30°C) inhibited starch metabolism by decreasing starch synthase activity due to thermal denaturation (Pravallika et al., 2020). Reduced grain endosperm starch content is a leading cause of reduced quality and yield in crops subjected to drought and heat (Worch et al., 2011). Similar to drought, heat stress decreases starch content but increases grain protein and mineral concentrations (Mariem et al., 2021). Heat stress reduced amylose content and partially altered the fine structure of amylopectin, indicating that the abnormal expression of the starch synthesizing enzymes is a key factor causing chalkiness (Nakata et al., 2017).

The changing climate is adversely affecting the nutritional quality in terms of mineral content and protein, which will impact human health (Mariem et al., 2021). Higher temperatures also decrease aroma quality in rice. Basmati rice had excellent aroma when grown under relatively cool temperatures in the afternoon (25–32°C) and night (20–25°C) and 70–80% humidity during the primordial and grain-filling stages (Singh et al., 2000). It is important to understand the physiological, biochemical and genetic mechanisms governing the response to combined heat and drought stress to develop strategies to improve stress tolerance.

#### 3.1.2 Physiological and genetic components of tolerance

Plants cope with drought and heat stress through cellular tolerance *via* metabolic homeostasis, osmotic adjustment, cellular membrane stability, oxidative stress management, production of stress proteins (e.g., late embryogenesis abundant proteins and HSPs) and secondary metabolites, and reducing fatty acid desaturation. Sucrose accumulated in the anthers of rice genotype Nagina 22 under combined drought and high-temperature stress (Li et al., 2015). Heat shock factors (HSFs) and HSPs showed differential upregulation in rice, with HSF7A upregulated under drought stress, *HSF2a* upregulated under heat stress, and HSP74.8, HSP80.2, and HSP24.1 upregulated under the combined stress (Piveta et al., 2020).

He et al. (2018) noted that a complex regulatory network mobilizes these defenses by involving upstream signaling molecules that transmit the stress signal *via* hormones, ROS, and nitric oxide (NO). Under drought and heat stress condition, overexpression of the gene Rab7 (*OsRab7*) improved tolerance in rice by high survival rate, relative water content, chlorophyll content, gas-exchange characteristics, soluble protein content, soluble sugar content, proline content, and activities of antioxidant enzymes (CAT, SOD, APX, POD) than that of the wild-type. In contrast, the levels of hydrogen peroxide, electrolyte leakage, and malondialdehyde of the transgenic lines were significantly reduced when compared to wild-type. Furthermore, the expression of four genes encoding reactive oxygen species (ROS)-scavenging enzymes (*OsCATA, OsCATB, OsAPX2, OsSOD-Cu/Zn*) and eight genes conferring abiotic stress tolerance (*OsLEA3, OsRD29A, OsSNAC1, OsSNAC2, OsDREB2A, OsDREB2B, OsRAB16A, OsRAB16C*) was significantly up-regulated in the transformed rice lines as compared to their expression in wild-type (El- Esawi and Alayafi, 2019).

Combined heat and drought stress studies have been undertaken on a few cultivars in rice, with one study identifying Nagina 22 as the only tolerant cultivar (Reshma et al., 2021). Therefore, systematic screening of rice germplasm and mapping populations are needed to identify and introgress QTL into elite cultivars. Genome-wide association studies can identify QTL/genes for dissecting the genetic basis of combined stress tolerance. The grain-filling stage is one of the most important phases that determine yield. Stay green traits can be used as an indicator of sustainable assimilate supply and stem reserve utilization to promote seed filling under stressful conditions (Abdelrahman et al., 2017). There is an immense need to identify plant species and genotypes tolerant to combined stresses (Zandalinas et al., 2018) and tailor genotypes with acceptable performance under combined drought and high-temperature stress for sustainable crop production.

### 3.2 Effect of combined drought and elevated CO_2_

#### 3.2.1 Physiological and genetic components of sensitivity

Rice requires 5000 L of water to produce 1 kg biomass and 3,000–5,000 L for 1 kilo grain (Mainuddin et al., 2020). Studies in controlled environment chambers showed that eCO_2_ reduced evapotranspiration, allowing photosynthesis to continue for 1–2 days longer than aCO_2_ under drought stress (**Supplementary Figure 1**). While the saturation point for CO_2_ is 500 ppm in rice, the down regulation of photosynthesis occurred beyond 900 ppm. In addition, eCO_2_ attenuated the canopy dark respiration. Dark respiration has physiological relevance, as the energy derived is used for plant growth and metabolism (Zou and Xu, 2021). The reduced stomatal aperture increased the canopy temperature due to the suppression of transpiration. Prolonged exposure to eCO_2_ also reduced the net photosynthetic rate. The resultant decrease or increase in yield will be location specific, influenced by regional temperatures. Drought stress increases ABA content, which affects CO_2_ intake. Drought stress also reduces the levels of RuBisCo large and small subunits at the proteomic level. Thus plants cannot harness all of the benefits of CO_2_ fertilization under drought stress (Perdomo et al., 2017).

Prolonged drought stress significantly decreases some core physiological traits. The eCO_2_ treatment increased RuBisCo activity by 17.5% compared to the aCO_2_ treatment. One study showed that eCO_2_ (700 ppm) treated plants under drought stress had a 40% lower CO_2_ exchange rate than drought-stressed plants under aCO_2_ (350 ppm). The Km of RuBisCo also decreased compared to irrigated and drought-stressed plants under aCO_2_. Plants raised in a CO_2_-enriched atmosphere had higher RuBisCo content and activity after ~20 days of drought stress, but this comparative advantage did not occur after ~30 days of stress. In this situation, eCO_2_ plants had inferior physiological traits. Prabnakorn et al. (2018) reported that rice production would suffer more under climate change events, where increases in CO_2_ cannot mitigate the adverse effects on rice productivity.

#### 3.2.2 Physiological and genetic components of tolerance

Rice grown under eCO_2_ has more tillers and higher grain yield (Cho and Oki, 2012). eCO_2_ increased biomass by 5.7% due to an increased leaf area index and leaf water potential in rice (Kumar et al., 2017). Certain simulation models have highlighted the significance of CO_2_ fertilization in assisting crops to withstand water deficits (Kang et al., 2021). A meta-analysis study on rice, wheat, and maize under increased CO_2_ levels and drought stress revealed that the CO_2_ component alone increased grain yield and starch content but decreased protein and mineral contents. The inevitable consequence of stomatal conductance for CO_2_ leads to loss of water, affecting the proportion of net photosynthesis to transpiration rate (i.e., transpiration efficiency), as a function of leaf anatomical features that determine the utilization of CO_2_ levels in the atmosphere (Ouyang et al., 2017). Under eCO_2_ (700 ppm), the imposition of drought stress had less effect on yield attributes than aCO_2_ and reduced water use by 10% (Shanker et al., 2022) Similarly, combined eCO_2_ and drought stress maintained canopy net photosynthesis by 6−12%. CO_2_ supply extended the maintenance of mid-day photosynthesis for a few days, which had an ameliorative effect on rice.

In rice, a soil matric potential of –40 kPa (~43% moisture) or below results in water deficit stress (Kumar et al., 2019). An eCO_2_ (550 ppm) treatment at a 2°C elevated temperature imparted intrinsic drought (–40 kPa) stress tolerance traits in aerobic rice genotypes (CR-143-2-2, APO, and CR Dhan 201), reducing antioxidant enzyme (SOD, POX, CAT) activities in leaves (Padhy et al., 2018). Drought stress also decreased the aboveground biomass and yield in IR72. However, an eCO_2_ (700 ppm) treatment maintained higher rice biomass and yield than aCO_2_ (350 ppm), with both CO_2_ regimes maintaining a comparable harvest index in corresponding treatments. Both CO_2_ regimes increased sucrose and reduced starch content in drought-stressed IR72, reducing grain quality. Plants raised under aCO_2_ conditions exposed to drought stress had more pronounced reductions (45%) in sucrose phosphate synthase activity (sucrose biosynthesis enzyme) than those raised under eCO_2_ (Wang et al., 2022).

Under drought stress, ABA acts as the primary regulator of stomatal closure, eCO_2_ delays the sunthesis of ABA. Crosstalk also occurs between these two components at the aquaporin level (Li et al., 2020). A brassinosteroid (BR) treatment ameliorated the ill-effects of drought stress by improving CO_2_ assimilation (Raghunath et al., 2021; Lakshmi et al., 2022). The induction of endogenous BR under drought stress might help accumulate carbon. A study on *d1* mutants for the Gα subunit (of heterotrimeric G protein complex) gene RGA1 (Rice Gα subunit 1) reported that Nipponbare and Taichung 65 had higher mesophyll conductance for CO_2_ than the wild type and likely had higher WUE and productivity under drought stress (Zait et al., 2021). Overexpression of the *OsEPF1* (Epidermal Patterning Factor 1) gene reduced stomatal density in rice, enhancing drought tolerance but compromising yield, which improved with 450–480 ppm CO_2_ supply. Such a plant type will benefit future climate scenarios with scant rainfall and elevated CO_2_ (Caine et al., 2019).

### 3.3 Effect of combined high temperature and eCO_2_

#### 3.3.1 Physiological and genetic components of sensitivity

Periods of high temperature and eCO_2_ concentration due to anthropogenic activities threaten rice production (**Supplementary Table 2**). eCO_2_ should enhance the photosynthetic rate, increasing total yield and productivity (Kant et al., 2012; Hasegawa et al., 2013) because CO_2_ is directly involved in major physiological processes such as photosynthesis and stomatal conductance. Rising temperatures reduce rice yield alone or in combination with eCO_2_ (Wang et al., 2020). A higher respiration rate and declining membrane thermostability reduce rice yield under high night temperature (HNT) conditions (Mohammed and Tarpley, 2010). The decreased membrane stability index in susceptible rice varieties under elevated temperature was related to the extent of lipid peroxidation by ROS (Das et al., 2014; Kumar et al., 2016).

The most sensitive stages to high-temperature stress in rice are booting, anthesis, and fertilization. Several studies have investigated the effect of high temperature and eCO_2_ concentrations in rice in growth or open-top chambers. The closed chamber experiments revealed that rice is highly susceptible to heat stress and heat-induced spikelet sterility (HISS) at flowering, resulting in yield losses. eCO_2_ cannot ameliorate yield losses due to the high temperature (Wang et al., 2018). Cai et al. (2016) and Wang et al., (2018, 2020) reported that rising temperatures decreased panicle number per unit area and spikelet number per panicle, decreasing rice yields; these effects escalated under eCO_2_. eCO_2_ alone exacerbates HISS as stomatal closure increases the canopy temperature, with a stimulatory effect on biomass production, but an increase in night temperatures counteracts this effect. Significant compromises in yield occur due to the higher respiratory cost of the increased biomass. Night respiration increased by 4–18 mg C hill^–1^ h^–1^ in rice genotypes under eCO_2_ and HNT at various crop stages before heading (Shanker et al., 2022).

The interactive effect of heat stress and eCO_2_ adversely impacts rice growth, development, and pollen viability (Mittler et al., 2012). Decreased anther dehiscence, poor pollen shedding, poor pollen grain germination on stigmas, and decreased pollen tube elongation led to spikelet sterility under heat stress. Raised night temperatures have more adverse effects than raised day temperatures due to deprived anther dehiscence, impaired pollination, abnormal pollen germination, and floret sterility (Das et al., 2014; Fahad et al., 2018). Floral sterility under high temperatures reduces sink demand due to the reduction in carbohydrate transfer from shoots to grain (Madan et al., 2012). Active selection and breeding for the eCO_2_ response and HNT-resilient rice are needed to compensate for yield losses.

Heat stress during the reproductive and grain-filling stage reduces rice yield by diminishing the proportion of fertile spikelets (Beena et al., 2018a), shortening the grain-filling period (Ahmed et al., 2015), and reduction in sink activity (Kim et al., 2011). Thus, elevated CO_2_ and high-temperature stress during flowering and early grain filling significantly reduce rice seed set and thousand-grain weight (Chaturvedi et al., 2017). eCO_2_ and high temperature also shorten the phenology of rice. Rice grain quality is reflected in parameters such as head and chalky rice rate, amylose and protein contents, and edible quality, as indicated by gel consistency. As CO_2_ and temperature increased, rice grain appearance initially declined but then improved (Liu et al., 2017). Exposure to high temperature during ripening causes abnormal morphology and grain discoloration in rice, probably due to reduced enzymatic activity related to grain filling, respiratory consumption of assimilation products, and decreased sink activity. Combined eCO_2_ and high-temperature stress significantly affects amylose content and gel consistency (**Supplementary Figure 2**). Madan et al. (2012) reported a slight decrease in amylose content and gel consistency in the sensitive genotype IR64, which carries one of two heat-sensitive alleles responsible for amylose accumulation during grain filling.

Soluble protein is the principal holder of plant nitrogen and an important index for measuring leaf aging. Liu et al. (2017) documented that soluble protein content did not vary widely across rice growth stages under eCO_2_ and high-temperature conditions. In another study, eCO_2_ stimulated grain production and starch accumulation but negatively affected nutritional traits such as protein and mineral contents (Mariem et al., 2021). The severity of eCO_2_ and high-temperature stress increases when the stress period coincides with flowering and grain filling and further intensified by high canopy temperatures associated with stomatal opening. Elevated CO_2_ combined with canopy warming affects plant C, N, and P ratios due to insufficient N uptake and allocation (Wang et al., 2019). The whole plant C/N ratio will remain unaffected if C assimilation and N absorption both increase under eCO_2_ and HNT conditions (Cheng et al., 2010).

#### 3.3.2 Physiological and genetic components of tolerance

Being a C3 crop, rice theoretically will benefit from the eCO_2_ fertilization effect, whereas the concomittent increase in temperature will negate the positive benefit of eCO_2_ (Chaturvedi et al., 2017). At the cellular level, the photosynthetic response to eCO_2_ will be greater at higher temperatures due to the reduction in RuBisCo activity. In addition, canopy photosynthesis will significantly increase with eCO_2_, which could negate the adverse effects of high-temperature stress on the C3 pathway (Kadam et al., 2014).

In contrast to high day temperature (HDT) stress, rice lacks an escape or avoidance mechanism under HNT stress (Bahuguna et al., 2014; Bahuguna et al., 2015; Hirabayashi et al., 2015). However, rice may have an enhanced ability to meet the increased carbon demand under increased night respiration, minimizing the negative impact of HNT on grain yield and quality (Impa et al., 2020). The usefulness of increased crop responsiveness to eCO_2_ under warmer nights has not been investigated. Bahuguna et al. (2022) reported that rice cultivars with significantly higher CO_2_ responsiveness could fix the additional carbon available under future scenarios.

FACE experiments revealed that eCO_2_ significantly reduced rice grain quality. However, newly developed heat-tolerant rice cultivars retained high grain quality under eCO_2_ (Usui et al., 2014), suggesting that current breeding efforts for heat tolerance will be useful for the projected climate change scenarios. Under climate change, the photosynthetic apparatus should be improved and some physiological responses such as stomatal conductance and transpiration rate should be maintained. The sensitivity of rice to HNT could be overcome by surveying germplasm to develop climate-resilient varieties for eCO_2_ responsiveness through marker development and genomic mapping (Silva et al., 2020; Bahuguna et al., 2022). **Supplementary Figure 2** shows the interactive effect of high temperature, and eCO_2._

### 3.4 Effect of combined salinity and drought stress

#### 3.4.1 Physiological and genetic components of sensitivity

Salinity and drought stress disrupt morphological features and physiological and biochemical processes in rice. While these stresses have their respective domains and scopes, drought and salinity stress often co-occur in natural field environments (Fan et al., 2015; Paul et al., 2019; Yadav et al., 2022). The severity and occurrence of combined drought and salinity stress are expected to increase with global environmental changes, which could have profound implications on the food supply. This combined stress is a major limiting factor for rice cultivation and productivity (Landi et al., 2017), triggering oxidative, osmotic, and temperature stresses leading to cellular dehydration and reduced cytosolic and vacuolar volume (Fan et al., 2015). ROS production under combined salinity and drought stress amplifies the damage to proteins, DNA, and membranes (Landi et al., 2017), reducing the photosynthetic rate and efficiency and inducing programmed cell death; thus reducing yields by more than 30% each year (Bhar, 2020).

Several studies have shown that drought and salt stress share similar initial plant responses, resulting in ion toxicity in the long term. Salinity and drought stress both cause physiological water deficits that affect all plant organs to varying degrees. However, plants react to hyper-ionic and hyper-osmotic stress under extended salt stress. Concomitantly large VPD also increases under drought stress. The effect of drought and salinity on photosynthesis ranges from restricted CO_2_ diffusion into chloroplasts, limited stomatal opening mediated by shoot and root-generated hormones and CO_2_ transport through the mesophyll, and changes in leaf photochemistry and carbon metabolism (Ma et al., 2020). The combined effect of drought and salinity at early stages (germination, seedling establishment, and tillering) delays transplantation (in rainfed lowlands) or crop establishment (in uplands) and stunts growth, resulting in poor stand establishment and ultimately reducing the number of panicles per unit area and panicle size. The combined stresses at the reproductive stage (panicle initiation, flowering, and grain filling) cause varying degrees of spikelet sterility and poor grain filling, with greater detrimental effects on grain yield (Ali et al., 2022).

#### 3.4.2 Physiological and genetic components of tolerance

Most drought and salt stress studies focus on roots and shoots, with measurements of physiological and genetic parameters (Qin et al., 2020; Hao et al., 2022). Among them, ABA plays an important role in plant responses to abiotic stresses (Zhao et al., 2021). The overexpression of *OsPYL5* can improve drought and salt tolerance through ABA-mediated processes (Ruiz et al., 2021). Secondary messengers such as Ca^2+^ and ROS can alleviate osmotic stress damage and improve drought and salt tolerance through ABA-dependent/independent pathways. In addition, H_2_O_2_ plays a vital role in stomatal closure through ABA-dependent and ABA-independent pathways (Chen et al., 2021). Under drought and salt stress, stress-response genes increase plant resistance by activating the associated proteins and accumulating protective metabolites. Downregulating the expression of *DST1* (*DROUGHT AND SALT TOLERANT 1*), *ABIL2* (ABL INTERACTOR-LIKE PROTEIN 2), and *HDA704* (histone deacetylase) positively regulates drought and stress tolerance in rice. *hda704* knockdown mutants exhibited susceptibility to drought and salinity stress. HDA704 imparts drought tolerance by promoting stomatal closure (Zhao et al., 2021). Shikimate pathway is known to be activated under abiotic stress conditions, such as drought and salinity, resulting in the accumulation of high levels of aromatic amino acids and related secondary metabolites (Francini et al., 2019). Overexpression of *OsSKL2* in rice increased tolerance to salinity, drought and oxidative stress by increasing antioxidant enzyme activity, and reducing levels of H_2_O_2_, malondialdehyde, and relative electrolyte leakage (Jiang et al., 2022).

### 3.5 Effect of combined salinity and submergence stress

The changing climate and resultant rise in sea water levels lead to unexpected spells of multiple abiotic stresses at different stages of paddy production. In coastal areas, increasing temperatures, erratic rainfall, and inundation of saline water due to sea-level rises can change the micro-environment in fields. Studies are limited in this arena for rice. Tolerant rice genotypes adapt to combined salinity and submergence due to the presence of well-developed constitutive aerenchyma and increased ethylene production and respiratory burst oxidase homolog (RBOH) signaling. RBOH-mediated ROS production resulted in the development of constitutive aerenchyma in a saline and flooding tolerant rice variety, Rashpanjor (Chakraborty et al., 2021). Chlorophyll fluorescence imaging identified tolerant varieties under combined salinity and partial submergence (Pradhan et al., 2018).

### 3.6 Effect of combined salinity and high temperature

**Figure 2:**
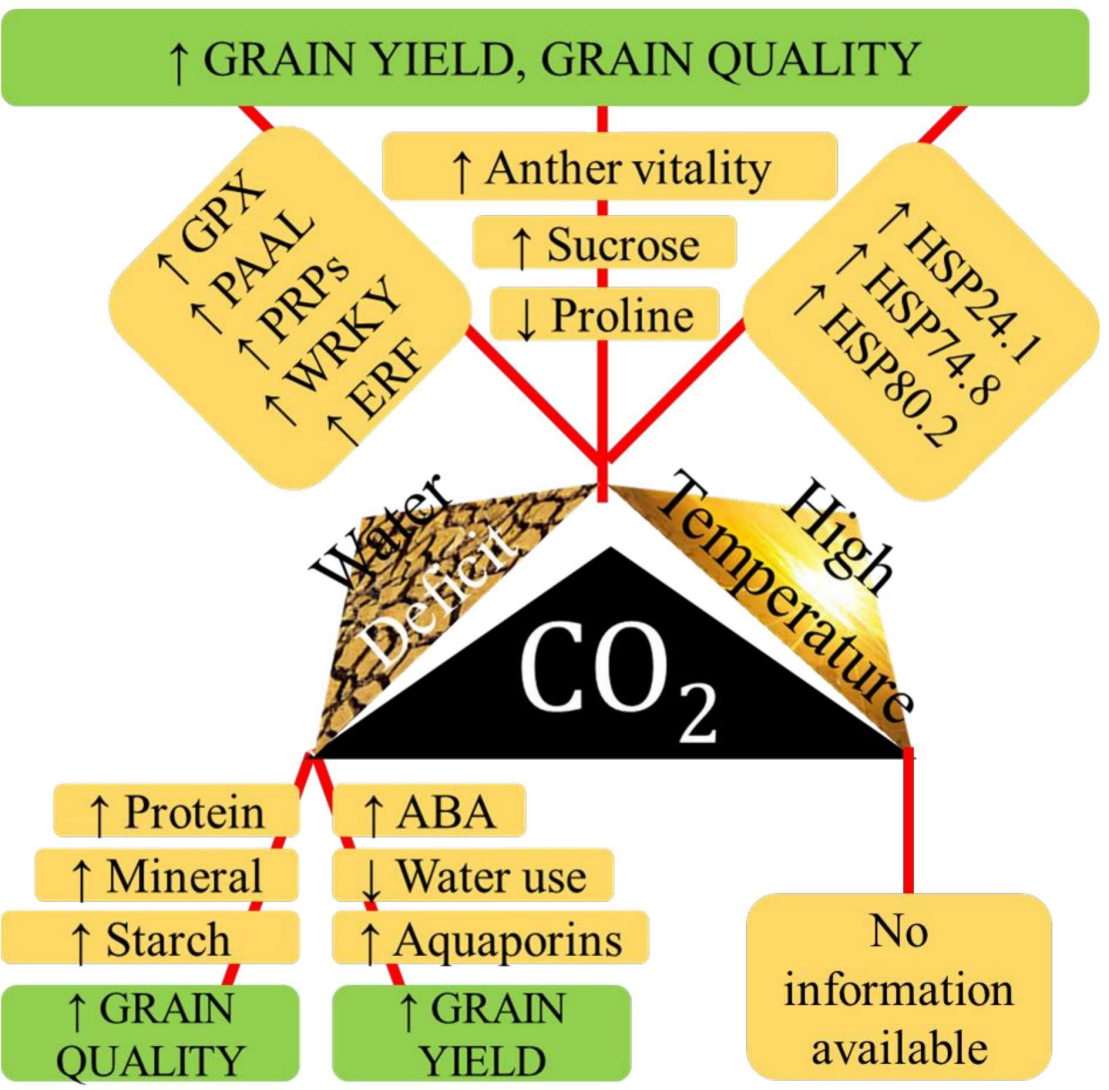
The physiological aspects of tolerance to various paired combinations of high temperature, water deficit stress and elevated CO2 with respect to grain yiled, and quality in rice.

High temperature and salinity in tropical coastal belts derail rice productivity. Exposure to salinity and high temperature, in combination or in tandem, changes rice growth patterns, defense mechanisms, reproduction, and survival functions, reducing shoot fresh weight, relative water content, photosynthetic pigments, and protein content and increasing proline and SOD activities. A saline-tolerant rice variety, YNU31-2-4, under combined high temperature and salinity stress, downregulated K^+^ transporter *OsHKT1*;*5* and upregulated *OsHSP18*, *OsP5CS*, and Na^+^/H^+^ antiporter *OsNHX* (Nahar et al., 2022). However, under combined stress condition Nagina-22 performed well than other genotypes in terms of proline content, cell membrane stability index, SOD activity, pollen viability, spikelet fertility, and yield per plant and lower lipid peroxidation and Na^+^/K^+^ ratio than susceptible genotypes (Ali et al., 2021). Combined effects of various abiotic stresses on physio-biochemical traits in rice is given in **Supplementary Table 2**. **Figure 2** shows the interactive effect of high temperature, eCO_2._ and drought.

## 4 Conclusion

Rice (*Oryza sativa* L.) is the staple food crop consumed by much of the world’s population. Projected rice statistics for 2021–22 estimated global production of 505.4 million tons, an increase of 1.9 million tons than previous year, mainly attributed to China, Bangladesh, South Korea, and Taiwan. Paddy is cultivated primarily in tropical climates, where water scarcity, high temperatures, salinity, and nutrient deficits can significantly reduce yields. Rapid fluctuations in environmental conditions can impact the adaptive ability of rice, further impairing its productivity. Various abiotic stresses affect seed germination, seedling establishment, shoot and root lengths, plant height, days to flowering, grain filling, maturity, and grain quality. Abiotic stresses during both vegetative and reproductive stage compromise panicle development and grain filling, impacting overall grain production and jeopardizing global food security. Genomics and QTL-based approaches have helped identify genes and loci responsible for abiotic stress tolerance in rice. Introgressing these newly identified molecular candidates can improve rice physiological growth under suboptimal conditions and stimulate reproductive development and grain production. However, further studies involving next-generation sequencing platforms and high-throughput phenotyping will help identify novel candidate genes responsible for regulating grain development in combined stress situations and pave the way for developing climate-ready crops.

## Author contributions

BR, RS and GK conceived and designed the study; GK prepared the figures; All authors review the literature, synthesize the data/material and draft the review; KS critically edited the manuscript; RS, MT, GK, DU, ST, CA, BS, BM and BR, helped in developing main andsupplementary tables. All authors have read and agreed to the published version of the manuscript.
