# Supplementary material for: Physiological and molecular implications of multiple abiotic stresses on yield and quality of rice
Source: Front Plant Sci. 2023 Jan 11;13:996514. doi: 10.3389/fpls.2022.996514 (PMC9874338; doi:10.3389/fpls.2022.996514)
Supplement: Supplementary file 1 [file DataSheet_1.docx]

**Supplementary tables:**

**Supplementary Table 1: Effect of various abiotic stress condition on physiological and biochemical traits in rice.**

| **Physiological and biochemical traits affected under various abiotic stress condition** | **References** |
| --- | --- |
| **Drought/water stress** | |
| Photorespiratory pathway forms excess amounts of superoxide radical, hydrogen peroxide and hydroxyl radicals. | Gill and Tuteja, 2010a |
| Chlorophyll loss due to decreased mesophyll potential | Sarwar et al., 2013 |
| Stomata closure, lowered turgor pressure, decreased CO_2_ uptake by the plant, and an increase influx of excess electrons for the generation of ROS | Gupta et al., 2020 |
| Drought stress causes an increase in Superoxide dismutase levels, as well as changes in catalase activity, an increase in Guaiacol peroxidase, and activation of the Ascorbate-Glutathione pathway. | Gill and Tuteja 2010b; Mishra and Panda, 2017; Laxa et al., 2019 |
| Build-up of osmoprotectants, such as proline, glycine betaine and soluble sugar. | Kumar et al., 2016; Per et al., 2017; Upadhyaya and Panda, 2019 |
| Reduced and impaired RuBisCo activity, a significant decline in the centers for O_2_ evolution and the photosystem, which inhibits the electron transport chain and subsequently causes PSII to become inactive | Mishra and Panda, 2017; Mishra et al*.*, 2018 |
| Increase in lipid peroxidation and protein denaturation | Ullah et al., 2018 |
| Membrane water exclusion cause lipid structure dislocation | Upadhyaya and Panda,2019 |
| Leaf rolling | Cal et al., 2019 |
| Activation of the ethylene phytohormone, brassinosteriod, and abscisic acid pathways | Gupta et al., 2020 |
| Reduction in stomatal conductance, transpiration rate and photosynthetic rate | Claeys and Inz´e, 2013; Lauteri et al., 2014; Estravis-Barcala et al., 2020; |
| **Submergence/Flash flooding** | |
| Rice varieties tolerant to flash flooding exhibit limited or no elongation during complete submergence, whereas deepwater rice varieties adopt greater elongation of leaf and culm | Luo et al, 2011 |
| Under complete submergence, photosynthesis is weakened, and plants exhaust the reserve carbohydrate and ultimately die | Luo et al, 2014 |
| Plants tended to utilize more photosynthates towards culm formation rather than leaf area development. | Voesenek and Bailey-Serres,  2015 |
| Stagnant flooding stress increases aerenchyma gas space and reduces root oxidase activity | Kuanar et al., 2017 |
| **Salinity** | |
| Increased osmotic potential and decreased water availability to plants cause cell membrane dehydration, decreased CO_2_ permeability, and restriction of the photosynthetic ETS due to intercellular space shrinkage. | Gadelha et al., 2021 |
| Cl- ion toxicity reduces root nitrate uptake, which inhibits photosynthetic rate. |  |
| Stomatal closure decreases CO_2_ supply and availability for carboxylation reactions. |  |
| Increased rate of salinity-induced leaf senescence |  |
| Changes in cytoplasmic structure and enzymatic activities |  |
| Low transpiration rate, reduced chloroplast activity, and impaired stomatal conductance | Gupta and Huang, 2014 |
| Reduced activity of enzymes involved in carbon fixation and antioxidants | Horie et al., 2012 Gupta and Huang, 2014 |
| Reduced photosynthetic pigments | Aref and Rad, 2012; Hariadi et al., 2015 |
| Reduction in Nitrogen balance index and  Chlorophyll content | Kakar et al, 2019 |
| Reduction in Fv/Fm ratio | Yang et al., 2020 |
| Increase in Superoxide dismutase (SOD), Peroxidase, Catalse (CAT), Glutathione reductase (GR), and Ascorbate peroxidase (APX) activity | Rajkumar et al., 2022 |
| **High temperature** | |
| Spikelet sterility increased | Yoshimoto et al., 2011 |
| Differential changes in proteome | Jagadish et al., 2011 |
| Lipid peroxidation, SOD activity  POD activity increased | Xue et al., 2012 |
| Pollen viability and germination reduced. | Das et al., 2014; Fahad et al., 2018 |
| Membrane stability index reduced. | Das et al., 2014; Kumar et al., 2016 |
| Sugar content in anther reduced. | Li et al., 2015  Shi et al., 2018 |
| Fv/Fm ratio reduced. | Coast et al., 2020 |
| **Elevated CO_2_ condition** | |
| Relative water content (RWC %), membrane stability index (MSI %), chlorophyll content, photosynthetic rate and TSS content were improved under elevated CO_2_. | Dwivedi et al., 2015 |
| Increased photosynthesis, leaf inter cellular CO_2_ concentration, water use efficiency and reduction in stomatal conductance, transpiration rate, accumulation of more soluble sugar and starch. | Dorneles et al., 2020 |
| Increase in peroxidise activity and reduction in super oxide dismutase activity | Senthil‑Nathan, 2021 |

**Supplementary Table 2. Physio-biochemical effects of multiple abiotic stresses in rice**

| **Physio-biochemical traits affected** | **Effect** | **Reference** |
| --- | --- | --- |
| ***Drought and temperature*** | | |
| Transpirational cooling | Reduction | Costa et al., 2021 |
| Protein catabolism | Increase | Jagadish et al., 2011 |
| Lipid peroxidation | Increase |  |
| PSII function | Reduction |  |
| Leaf gas exchange | Reduction | Perdomo et al., 2015; 2016 |
| Water use efficiency | Reduction |  |
| Growth and biomass accumulation | Reduction |  |
| Panicle number | Reduction | Mukamuhirwa et al., 2019 |
| Anther dehiscence and pollen germination | Reduction | Rang et al., 2010; 2011 |
| Peduncle length | Reduction |  |
| Pollen number | Reduction |  |
| Spikelet sterility | Increase |  |
| Starch biosynthesis | Inhibition | Costa et al., 2021 |
| Protein biosynthesis | Reduction |  |
| Amylose content | Reduction |  |
| structure of amylopectin | Alteration |  |
| Grain chalkiness | Increase | Lawas et al., 2018 |
| Grain protein and mineral content | Increase | Mariem et al., 2021 |
| LEAs and HSPs accumulation | Increase | Piveta et al., 2020 |
| Fatty acid desaturation | Reduction |  |
| Sucrose accumulation | Increase | Li et al., 2015 |
| ***Drought and elevated CO_2_*** | | |
| Calvin cycle of photosynthesis | Reduction | Shankar et al., 2022 |
| Canopy dark respiration | Reduction |  |
| Stomatal aperture | Reduction |  |
| Transpirational cooling | Reduction |  |
| Canopy temperature | Increase |  |
| RuBisCo large and small subunits | Reduction |  |
| CO_2_ exchange rate | Reduction |  |
| Productivity | Reduction | Prabnakorn et al., 2017 |
| Protein and mineral content in grains | Reduction | Ouyang et al., 2017 |
| Canopy net photosynthesis | Improvement |  |
| Maintenance of mid-day photosynthesis | Improvement |  |
| Synthesis of ABA | Delayed | Li et al., 2020 |
| ***High temperature and eCO_2_*** | | |
| Grain filling | Reduction |  |
| Panicle number | Reduction | Wang et al., 2020 |
| Spikelet number per panicle | Reduction |  |
| Thousand-grain weight | Reduction | Chaturvedi et al., 2017 |
| Grain appearance | Improvement | Liu et al., 2017 |
| C, N, and P ratios in grain | Alteration | Wang et al., 2019 |
| Whole plant C/N ratio | No change | Cheng et al., 2010 |
| ***Salinity and drought*** | | |
| ROS production | Increase | Landi et al., 2017 |
| Damage to proteins, DNA and membranes | Increase |  |
| Programmed cell death | Increase | Bhar et al., 2020 |
| Photosynthetic rate and efficiency | Reduction | Wu et al*.,* 2019 |
| Grain filling | Reduction |  |
| Grain yield | Reduction |  |
| ***Salinity and submergence*** | | |
| Aerenchyma formation | Increase | Ray et al., 2021 |
| Respiratory burst oxidase homolog (RBOH) signaling | Increase |  |
| Ethylene production | Increase |  |
| ***Salinity and high temperature*** | | |
| K^+^ transporter *OsHKT1;5* | Downregulation | Nahar et al., 2022 |
| Upregulated *OsHSP18*, *OsP5CS* | Upregulation |  |
| Na^+^/H^+^ antiporter *OsNHX* | Upregulation |  |

**Supplementary Figures:**


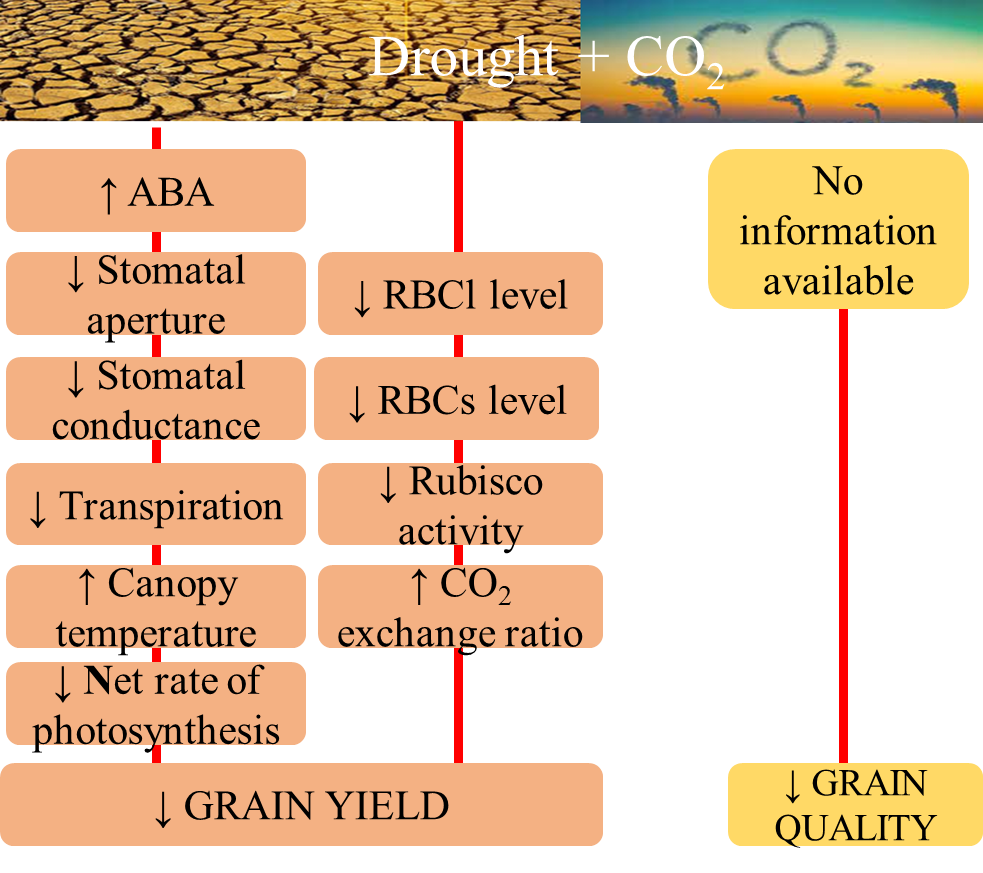


**Supplementary figure 1. The physiological aspects of sensitivity to combined drought and elevated CO_2_ treatment in rice with respect to grain yield.**


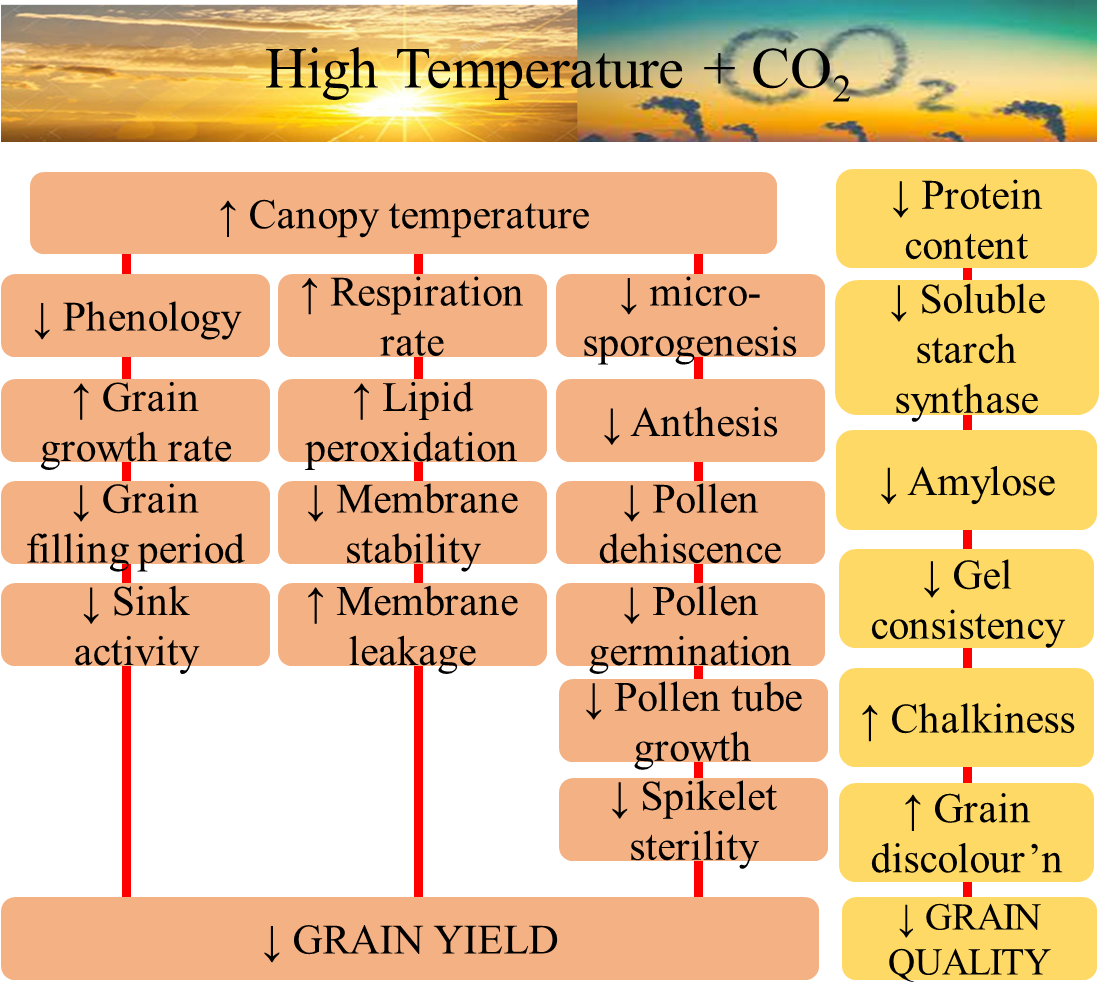


**Supplementary figure 2. The physiological aspects of sensitivity to high temperature stress in combination with elevated CO_2_ treatment in rice with respect to grain yield, and quality.**
